# The complete mitochondrial genome of water deer in Liaoning, China

**DOI:** 10.1080/23802359.2020.1719936

**Published:** 2020-01-29

**Authors:** Zongzhi Li, Zhirong Zhang, Shuhui Mi, Jianping Wu, Tao Xu, Zhensheng Liu, Liwei Teng

**Affiliations:** a College of Wildlife and Protected Area, Northeast Forestry University, Harbin, China;; b Forestry Development and Service Center of Benxi, Benxi, China;; c Key Laboratory of Conservation Biology, National Forestry and Grassland Administration, Harbin, China

**Keywords:** Complete mitochondrial genome, *Hydropotes inermis*, Cervidae

## Abstract

We determined the whole mtDNA genome of the water deer (*Hydropotes inermis)* in Benxi, Liaoning. The total length of the complete mitochondrial genome is 16,355 bp and it consists of 13 protein-coding, 22 tRNA, rRNA genes, and 1 control region (CR). Two overlaps among the 13 protein-coding genes were found: ND4L/ND4 and ND5/ND6. The CR is 928 bp in length. The nucleotide composition is 30.52% A, % 33.38 T, 22.77% G, and 13.32% C.

There are two subspecies of water deer (*Hydropotes inermis*) which are naturally distributed in China (*H. i. inermis*) and Korea (*H. i. argyropus*), respectively (Grubb 2005; IUCN 2011). Water deer was widely distributed in Liaodong Peninsula, the North China Plain, and the Yangtze River in China in the history, but now mainly distribute in Jiangsu, Zhejiang, Jiangxi, Hubei, Fujian and Jilin province (Sheng 1992; Ma and Zhang 2009; Li et al. 2019)

The complete mitochondrial genome of the water deer was sequenced using muscle tissue gathered at Liaoning, China (123°56′23.33″E, 41°23′06.65″N), and stored in College of Wildlife and Protected Area, Northeast Forestry University (No. WD190617). The sequence was submitted to the GenBank with the accession number MN746502.

The total length of the mitogenome is 16,355 bp, with a base composition of 30.52% A, 33.38% T, 22.77% G, 13.32% C. The mitogenome consists of 13 protein-coding genes, 2 rRNA genes (12S rRNA and 16S rRNA), 22 tRNA genes, and 1 control region (CR). The total length of the 13 protein-coding genes is 11,384 bp long, which corresponds to 69.61% of the mitochondrial genome sequence length. All of the 13 protein-coding genes are encoded on the same strand except for ND6 in the light strand. Except for ND2, ND3 and ND5 (ATA start codon), ND4L (GTG start codon) and ATP6 (ATC start codon), the remaining 8 protein-coding genes initiate with ATG (COX1, COX2, COX3, ND1, ND4, ND6, CYTB, ATP8). The total length of all tRNA genes is 1,534 bp long, and ranging from 61 bp (tRNA-Ser) to 76 bp (tRNA Leu (UAA)). Lengths of two rRNA genes and control region are 958 bp (12 s rRNA), 1569 bp (16S rRNA) and 928 bp (control regions), respectively.

The phylogenetic relationship of the water deer was inferred using the Neighbor-Joining method (Saitou and Nei 1987) and conducted in MEGA7 (Kumar et al. 2016). The consequence of the tree revealed that the phylogenetic relationship of the water deer in our study is very closed to *Rucervus duvaucelii branderi* (MG788693.1) and grouped with the *Hydropotes inermis argyropus* (KP203884.1), *Hydropotes inermis* (EU315254.1) (Figure 1).

**Figure 1.**
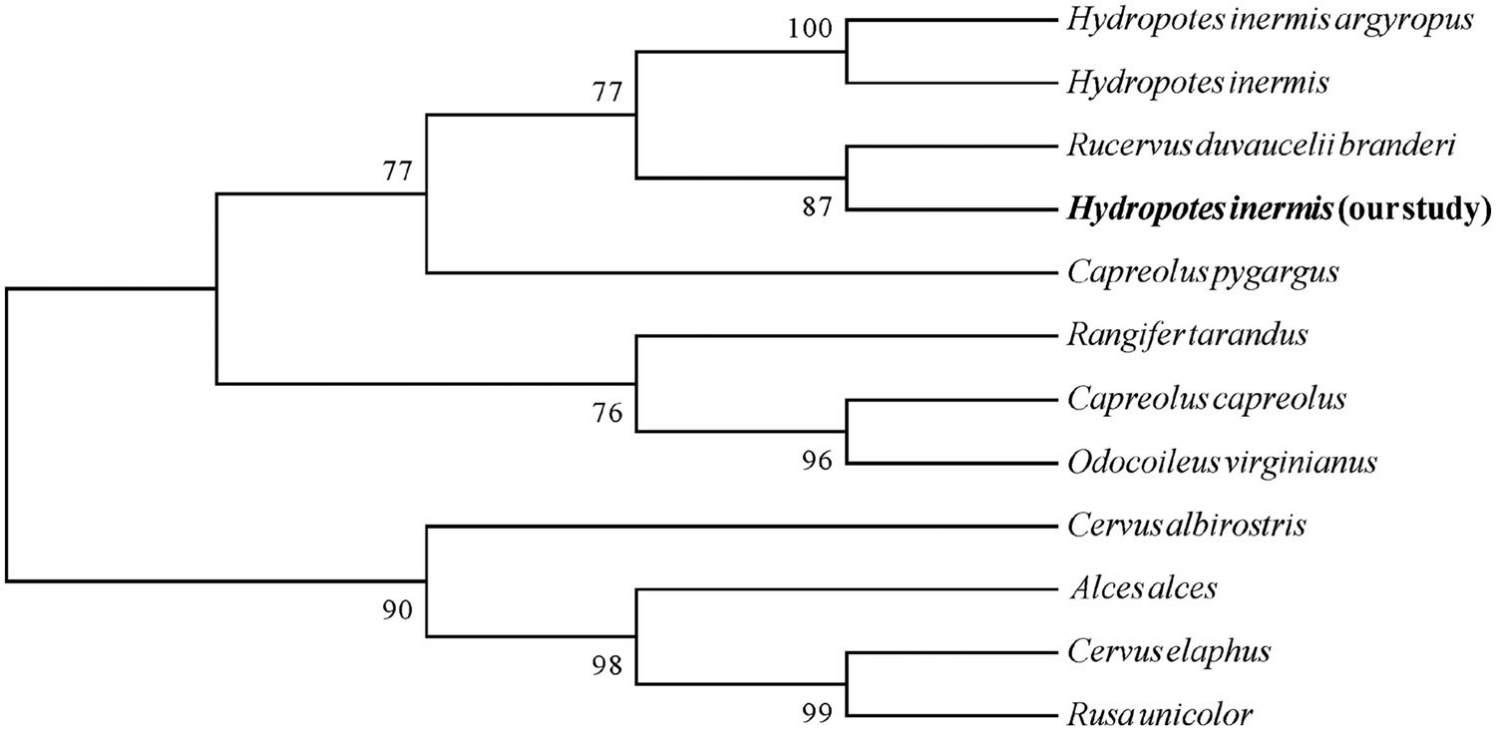
The phylogenetic tree generated by the Maximum Likelihood method based on complete mitogenomes of 11 species/subspecies in Artiodactyla: Cervidae. GenBank accession numbers: *Hydropotes inermis argyropus* (KP203884.1), *Hydropotes inermis* (EU315254.1), *Rucervus duvaucelii branderi* (MG788693.1), *Capreolus pygargus* (KJ681495.1), *Rangifer tarandus* (KM506758.1), *Capreolus capreolus* (KJ681489.1), *Odocoileus virginianus* (JN632672.1), *Cervus albirostris* (HM049636.1), *Alces alces* (JN632595.1), *Cervus elaphus* (KP172593.1) and *Rusa unicolor* (KX156946.1).
